# Alcohol in the Causation of Disease

**Published:** 1902-01-04

**Authors:** 


### ALCOHOL IN THE CAUSATION OF DISEASE.

Speaking recently at the Polyclinic, Professor Sims Woodhead formulated the part played by alcohol as a factor in the causation of disease. He says that alcohol is rapidly absorbed from the alimentary canal, and that the promptness with which it is absorbed and diffused means that it reaches the tissues practically unchanged, and that these tissues are exposed to the action of alcohol in an essentially unaltered condition. At a later stage the alcohol becomes oxidised. As showing the influence of alcohol on the protoplasm of the tissues, he instances its effect upon the cells of the central nervous system. Here it can be demonstrated that early and definite changes occur both in the cell and in its dendritic processes; changes which are not peculiar to alcohol, but are practically identical with those produced by the waste products of cell activity and by various other toxins. As showing the effects of alcohol upon connective tissue, he says that when circulating it appears to stimulate the endothelial cells around the small blood vessels, with the result that these cells undergo proliferation and ultimately new fibrous tissue is produced. This explains the so-called cirrhoses which mark the course of chronic alcoholism, and as alcohol when absorbed from the alimentary canal is conveyed by the portal vein directly to the liver, it is easy to understand why this organ is so frequently, and in so marked a degree, the site of cirrhotic change. Similar effects may be frequently detected in other organs and are common in the blood vessels. There is indeed, says Professor Woodhead, good reason to believe that alcohol may by itself and apart from other influences be responsible for a widespread arterio-sclerosis. The production of fatty changes in the tissue cells is another very common expression of the action of alcohol. This is seen in the nerve cells of the cerebral cortex in chronic drunkards, and is a frequent event in the cardiac muscle. It is, however, more especially in the liver that fatty changes attain their most prominent degree. But apart from the production of appreciable organic changes disturbance of function may take place, as is seen in the heart. One of the most important directions in which the function of the heart is disturbed by alcohol is that the ventricular systole is rendered incomplete. In health the contraction of the cardiac muscle ensures at each systole the practical obliteration of the ventricular cavity. But under the influence of alcohol the ventricular systole is not quite complete. A certain mass of blood remains and thus increases diastolic pressure. "The imperfect systole means that the muscular wall of the ventricle is not brought into a position where it can render effective support to the semilunar valves. Hence, under the repeated strain, these may yield to an extent sufficient to permit some measure of regurgitation. The regurgitant stream falling upon the ventricular wall during diastole takes the] muscular tissue at a disadvantage, and dilatation of the cavity of the ventricle is an inevitable consequence." At least so says Professor Woodhead. Still another serious effect of alcohol is that it interferes with leucocytic activity, thus leading to diminution of the resisting power to disease-producing agencies. All these considerations then must be borne in mind in an endeavour to fix the therapeutic value of alcohol. Whatever advantages it may have, as for example by dilating peripheral arterioles and lowering arterial tension, it must be remembered that along with these there are certain risks and penalties.

